# Neuroendocrine Disturbances in Huntington's Disease

**DOI:** 10.1371/journal.pone.0004962

**Published:** 2009-03-25

**Authors:** Nadine Saleh, Stéphane Moutereau, Alexandra Durr, Pierre Krystkowiak, Jean-Philippe Azulay, Christine Tranchant, Emmanuel Broussolle, Françoise Morin, Anne-Catherine Bachoud-Lévi, Patrick Maison

**Affiliations:** 1 Centre de référence maladie de Huntington, AP-HP, Groupe Henri-Mondor Albert-Chenevier/Pitié Salpétrière, Créteil, Paris, France; 2 Service Pharmacologie Clinique, AP-HP, Groupe Henri-Mondor Albert-Chenevier, Creteil, France; 3 Universite Paris 12, Faculte de Medecine, Creteil, France; 4 INSERM, Unite U955, Equipe 1, Creteil, France; 5 Service de Biochimie, AP-HP, Groupe Henri-Mondor Albert-Chenevier, Creteil, France; 6 INSERM U289, Department of Genetics, Cytogenetics and Embryology, Pitié Salpétrière, Paris, France; 7 Neurology Department, CHU Amiens, Amiens, France; 8 APM, CNRS, La Timone Hospital, Marseille, France; 9 Neurology Department, Hôpitaux Universitaires, Strasbourg, France; 10 Neurology Department, Pierre Wertheimer Neurological Hospital, Lyon, France; 11 EFS Ile de France, Bobigny, France; 12 Ecole normale Supérieure, Département d'études Cognitives, Paris, France; 13 Unité de recherche clinique, AP-HP, Groupe Henri-Mondor Albert-Chenevier, Creteil, France; Tokyo Medical and Dental University, Japan

## Abstract

**Background:**

Huntington's disease (HD) is a severe inherited neurodegenerative disorder characterized, in addition to neurological impairment, by weight loss suggesting endocrine disturbances. The aims of this study were to look for neuroendocrine disturbances in patients with Huntington's disease (HD) and to determine the relationship with weight loss seen in HD

**Methods and Finding:**

We compared plasma levels of hormones from the five pituitary axes in 219 patients with genetically documented HD and in 71 sex- and age-matched controls. Relationships between hormone levels and disease severity, including weight-loss severity, were evaluated. Growth hormone (GH) and standard deviation score of insulin-like growth factor 1 (SDS IGF-1) were significantly higher in patients than in controls (0.25 (0.01–5.89) vs. 0.15 (0.005–4.89) ng/ml, *p* = 0.013 and 0.16±1.02 vs. 0.06±0.91, *p* = 0.039; respectively). Cortisol was higher (*p* = 0.002) in patients (399.14±160.5 nmol/L vs. 279.8±130.1 nmol/L), whereas no differences were found for other hormone axes. In patients, elevations in GH and IGF-1 and decreases in thyroid-stimulating hormone, free triiodothyronine and testosterone (in men) were associated with severity of impairments (Independence scale, Functional score, Total Functional Capacity, Total Motor score, Behavioral score). Only GH was independently associated with body mass index (β = −0.26, *p* = 0.001).

**Conclusion:**

Our data suggest that the thyrotropic and in men gonadotropic axes are altered in HD according to the severity of the disease. The somatotropic axis is overactive even in patients with early disease, and could be related to the weight loss seen in HD patients.

## Introduction

Huntington's disease (HD) is a rare, autosomal dominant, neurodegenerative disorder resulting from expansion of a CAG repeat within the IT15 huntingtin (htt) gene on chromosome 4p [1]. Mutant huntingtin protein produced in the cytoplasm forms nuclear aggregates, which induce neuronal degeneration, most notably in the cerebral cortex and striatum [2]. Progressive neuronal death occurs in the tuberal nucleus of the lateral hypothalamus in patients [2]–[7] and transgenic mouse models [7]. This lateral hypothalamic abnormality would be expected to affect the function of most of the pituitary axes (as shown in other neurodegenerative diseases where neuroendocrine alterations occur [8]), potentially modifying the basal levels of GH, TSH, ACTH, LH, FSH, and prolactin, at least at late in the course of HD. However, the few available data on neuroendocrine function in HD are conflicting.

Basal GH levels were higher in HD patients than in controls in one study [9] and similar in another [10]. GH release in response to bromocriptine increased [11] or remained unchanged [10]. Excessive GH release was noted in response to arginine infusion or falling glucose levels [12], [13]. Prolactin levels were decreased [14], [15], increased [11], or unchanged [10], [16]. Cortisol levels were high [17], [18]. Thyroid stimulating hormone (TSH) was similar in patients and controls [19], and testosterone was decreased in male patients [20]. The discrepancies between axes and studies may be related not only to methodological factors, but also to differences across axes regarding the mechanisms that influence hormone production. Each study focused on a single pituitary axis, which precluded comparisons of axes within the same cohort. In addition, the number of patients was small [15], [20] (7 to 42), some studies had no control group, and the neuroendocrine effects of neuroleptic treatment were not always taken into account. Thus, available studies fail to provide a comprehensive picture of neuroendocrine function in patients with HD.

In other neurodegenerative diseases, neuroendocrine alterations are either independent from the phenotype, being directly induced by the disease process, or related to the phenotype (as a cause or a consequence) [8]. Neuroendocrine alterations in HD may develop over time in relation to the phenotype, as a result of advanced neuronal degeneration (e.g., causing atrophy of the pituitary gland) and progression of the disease. Alternatively, they may occur early on, in relation to the disease process, i.e., to proximal effects of the mutant huntingtin protein. The effects of the many protein-huntingtin interactions reported to date remain to be evaluated but may include selective disturbances in the hypothalamo-pituitary axes [21], [22]. If such is the case, the neuroendocrine disturbances would be expected to occur early according to the presence of mutant huntingtin protein from birth.

Weight loss is a characteristic feature of HD [23], [24], in addition to the neurological symptoms (chorea, cognitive impairment, and behavioral disturbances) [3]. Weight loss was first thought to be a consequence of increased energy consumption due to chorea. However, it is now acknowledged that weight loss can develop despite minimal involuntary movements [23], [25]. A recent study demonstrated a direct link between weight loss and CAG repeat length [26]. As well, endocrine abnormalities that influence weight [27] may cause the weight loss seen in HD.

We conducted a multicenter case-control study of the five anterior pituitary axes (somatotropic, thyrotropic, corticotropic, gonadotropic, and prolactin production) in a large cohort of patients with HD and in age- and sex-matched controls. Our primary objective was to look for neuroendocrine disturbances in patients with Huntington's disease (HD). Our secondary objective was to evaluate relationships linking plasma hormone levels, disease severity, and severity of weight loss.

## Results

The clinical characteristics of the patients and their controls are reported in Table 1. The patient cohort covered a broad age range (27–85) and was evenly distributed between males (n = 110) and females (n = 108). Disease severity was mild to moderate in most patients (31% stage I, 38% stage II, 23% stage III, and 9% stage IV–V), the number of CAG repeats was high (38 to 61), and mean age at symptom onset was 43.6 years ±10.5. Neuroleptics were used by 121 (56%) patients (40% typical neuroleptics, 59% atypical neuroleptics, and 1% both), antidepressants by 61% of patients, and tranquillizers by 37% of patients.

**Table 1:** Baseline characteristics of the patients with Huntington's disease and their age- and sex-matched controls.

	Patients (n = 217)	Controls (n = 71)
Age, years	52.23±10.52 (27–85)*	56.3±13.0 (26–84)
Females, n (%)	107 (49)	35 (49)
Weight, kg	65.32±12.75 (40–103)*	71.0±14.0 (47–105)
Height, m	1.68±0.09 (1.47–1.92)	1.69±0.10 (1.49–1.98)
Body mass index, kg/m^2^	22.8±3.52 (15.04–39.56) ***	24.7±4.24 (17.67–34.81)
Age at onset, years	43.6±10.5 (22–75)	-
(CAG) repeat length	45.0±3.8 (38–61)	-
UHDRS Functional assessment		
Functional score	32.2±6.4 (24–50)	-
Independence scale	78.0±16.7 (10–100)	-
TFC score	7.9±3.4 (0–13)	-
UHDRS Behavioral score	17.4±12.2 (0–61)	-
UHDRS Motor score	41.3±23.0 (1–110)	-

Data are means±SD (range) or % (n).

TFC, Total Functional Capacity; UHDRS, Unified Huntington's Disease Rating Scale.

* *p*≤0.05.

** *p*≤0.01.

*** *p*≤0.005.

Mean BMI and weight were significantly lower in the patients (22.8±3.5 and 65.32±12.75 kg, respectively) than in the control group (24.7±4.2, *p* = 0.004 and 71±14 kg, respectively) (*p* = 0.012).

### Somatotropic axis

Mean plasma GH level was significantly higher in HD patients than in controls (0.25 ng/ml [0.01–5.89] vs. 0.15 ng/ml [0.005–4.89], *p* = 0.04). The difference remained significant after adjustment for potential confounders including age, sex, BMI, and neuroleptic treatment (*p* = 0.017) (Table 2). Plasma GH was higher in patients who did not taking neuroleptics (n = 96, 0.38 ng/ml [0.01–10.24]), compared to their matched controls (n = 58, 0.14 ng/ml [0.004–4.57], p = 0.001) and to HD patients who was taking neuroleptics (n = 121; 0.18 ng/ml [0.01–3.16], *p*<0.001).

**Table 2:** Comparison of plasma hormone levels in patients with Huntington's disease patients and their age- and sex-matched controls.

	Plasma hormone levels	Patients (n = 217)	Controls (n = 71)	*p* values┬
Somatotropic	GH, ng/ml	0.25 (0.01–5.89)	0.15 (0.005–4.89)	0.017
	IGF-1, µg/L	154.45±49.20	142.35±41.66	0.042
	SDS IGF-1	0.16±1.02	0.06±0.91	0.039
	IGFBP3, µg/ml	4.95±0.98	4.99±1.05	NS
Corticotropic	ACTH, ng/L	7.9 (1.23–50.1)	7.06 (1.73–28.9)	NS
	Cortisol, nmol/L	399.14±160.5	279.8±130.0	0.002
Thyrotropic	TSH, mIU/L	1.36 (0.25–7.6)	1.39 (0.23–8.12)	NS
	FT4, pmol/L	15.74±3.3	16.42±2.04	NS
	FT3, pmol/L	4.71±1.58	4.75±0.52	NS
Gonadotropic (males only) (110 cases and 36 controls)	LH, IU/L	3.92 (1.44–10.7)	4.81 (1.28–18.2)	NS
	FSH, IU/L	5.31 (1.65–16.9)	6.39 (1.69–24.0)	NS
	Testosterone, nmol/L	15.62 (4.67–39.81)	14.18 (4–30.69)	NS
Prolactin	Prolactin, µg/L	15.39 (2.08–114.8)	8.65 (2.51–29.51)	NS

Data are arithmetic means±SD or geometric means (95% confidence interval). Adjustment was done on age, sex, BMI, neuroleptic treatment, and IGFBP-3 when these parameters were significant in the univariate analysis. GH, growth hormone; IGF-1, insulin-like growth factor-1; SDS-IGF-1, standard deviation score of IGF-1; IGFBP3, insulin-like factor binding protein-3; ACTH, adrenocorticotropic hormone; TSH, thyroid stimulating hormone; FT4, free total thyroxine; FT3, free triiodothyronine; LH, luteinizing hormone; FSH, follicle-stimulating hormone; NS, not significant.

┬ P value of the multivariate model.

Plasma IGF-1 and IGF-1SDS values were significantly higher in the HD patients than in the controls (154.45±49.20 µg/L vs. 142.35±41.66 µg/L, *p* = 0.042 and 0.16±1.02 vs. 0.06±0.91, *p* = 0.039; respectively). GH was the only pituitary hormone whose levels were significantly different between patients with early-stage HD (stage 1 or 2) and controls (*p* = 0.05), suggesting early dysfunction of the somatotropic axis.

### Corticotropic axis

ACTH concentrations were not significantly different between the patients (n = 219) and the controls (n = 71) (Table 2). However, plasma cortisol was higher in HD patients (399.14±160.5 nmol/L) than in controls (279.81±130.05 nmol/L, *p*<0.001). This difference persisted after adjustment for age, BMI, neuroleptics, and ACTH (*p* = 0.002). Plasma cortisol was higher in HD patients who were not taking neuroleptics than in their matched controls (409.49±152.45 nmol/L vs. 289.45±130.32 nmol/L, *p* = 0.001).

### Thyrotropic axis

As shown in Table 2, none of the thyrotropic axis hormones differed significantly between the HD patients and the controls group in the multivariate analysis.

### Gonadotropic axis in men

Since information on menopausal and menstrual cycle phase at the time of blood sampling for hormone assays was not available, we neglected the results in women and focused on those of men. LH, FSH, and testosterone concentrations showed no significant differences between all male HD patients and all male controls (Table 2).

### Prolactin

Prolactin levels were significantly higher in the patients than in the control group (15.39 µg/L [2.08–114.8] vs. 8.65 µg/L [2.51–29.51], *p*<0.001) (data not shown). Prolactin level elevation is a well-known effect of neuroleptics. HD patients who were taking neuroleptics (n = 121) had significantly higher prolactin levels, compared to the age-sexe matched controls (n = 66; 21.98 [2.81–186.2] µg/L vs. 8.27 [2.75–25.11] µg/L, *p*<0.001) and to the HD patients who were not taking neuroleptics (n = 96; 9.24 [2.51–33.95] µg/L, *p*<0.001). There was no difference in prolactin levels between HD patients who were not taking neuroleptics (n = 96; 9.24 µg/L [2.51–33.97]) and their matched control group (n = 58; 8.23 µg/L [2.95–22.90], not significant). After adjustment for neuroleptic use, prolactin levels were not significantly different between the patients and their matched controls (Table 2). Because the difference between sexes on the levels of prolactin, we evaluated prolactin levels separately for males and females. Analyses of gender-matched populations yielded similar results (data not shown).

### Relationships between disease severity and plasma hormone levels

GH (Table 2 and 3) was the only pituitary hormone that increases significantly both in HD patients compared to controls and across disease stages. IGF-1 increased with the severity of the functional and motor impairments (Table 3). These relationships remained significant in the multivariate analyses adjusting for age, sex, neuroleptics, IGFBP3 for IGF-1, and BMI for GH.

**Table 3:** Relationship between disease severity and pituitary axis function.

Clinical features	GH	IGF-I	ACTH	Cortisol	TSH	FT3	FT4	FSH†	LH†	TT†	Prolactin┤
Independence Scale	−0.22***	−0.14**	−0.007	−0.09	0.17**	0.19**	−0.05	−0.02	−0.10	0.25**	−0.05
Functional score	0.17**	0.15***	−0.04	0.02	−0.19**	−0.22***	0.06	0.03	0.07	−0.29***	0.06
TFC score	−0.21***	−0.13**	−0.02	−0.01	0.21***	0.19**	−0.02	0.02	−0.03	0.25**	−0.02
Total Motor score	0.15**	0.10*	0.07	0.06	−0.09	−0.23***	−0.07	−0.05	0.01	−0.18*	0.02
Behavioral score	0.03	−0.001	−0.04	−0.09	−0.12*	−0.01	0.009	0.01	0.04	−0.20**	−0.02

Data are *β* coefficient*s* of the linear regression. The multivariate linear regression model adjusted for age, sex, neuroleptic treatment, IGFBP3 when IGF-1 was in the model, and BMI when GH was in the model.

* *p*≤0.10.

** *p*≤0.05.

*** *p*≤0.01.

GH, growth hormone; IGF-1, insulin-like growth factor-I; ACTH, adrenocorticotropic hormone; TSH, thyroid stimulating hormone; FT3, free triiodothyronine; FT4, free total thyroxine; LH, luteinizing hormone; FSH, follicle-stimulating hormone; TT, testosterone; TFC, total functional capacity.

† Only males were included (110 patients).

┤ Only patients not taking neuroleptics were included (96 patients).

Decreases in testosterone levels in men and in TSH and FT3 in all patients occurred in parallel with disease severity as assessed by functional scales, the motor scale and, for testosterone in men, the behavioral score (Table 3).

Consistent with these results, in a multivariate analyze, the cognitive impairment, measured in 98 patients, was also related to the decrease in Testosterone in men and in FT3 and to the increase in IGF-1. (Data not shown)

### Relationship between body mass index and hormone plasma levels

BMI showed significant negative relationships (Table 4) with GH, cortisol, CAG repeat number, and motor UHDRS. These relationships remained significant after adjustment for age and sex. No other hormone was related to BMI (Table 4), including testosterone in men (data not shown). In the multivariate analysis, only GH was significantly related to BMI (β = −0.26, R^2^ = 0.12, *p* = 0.001) independently from age, gender, motor UHDRS, CAG repeat number, and cortisol (Table 4).

**Table 4:** Relationship between body mass index and clinical and hormonal disturbances.

Clinical and hormonal	BMI	BMI
features	Adjusted Analyses^1^	Multivariate Analysis^2^
GH	−0.27***	−0.21***
IGF-1	−0.14	-
ACTH	0.09	-
Cortisol	−0.16**	−0.13
TSH	−0.03	-
FT3	0.11	-
FT4	−0.04	-
Age at symptom onset	0.10	−0.01
CAG repeat number	−0.21*	−0.16
UHDRS motor score	−0.20***	−0.16
CAG repeat number	−0.21*	−0.16
UHDRS motor score	−0.20***	−0.16

Data are *β* coefficients (*p* value) of the linear regression.

1 adjusted for age and sex.

2 multivariate analysis including age, sex, and parameters significant in the univariate analysis.

* *p*≤0.10.

** *p*≤0.05.

*** *p*≤0.01.

BMI, body mass index; GH, growth hormone; IGF-1, insulin-like growth factor-I; ACTH, adrenocorticotropic hormone; TSH, thyroid stimulating hormone; FT4, free total thyroxine; FT3, free triiodothyronine.

Because a causal relationship between chorea and weight loss has been suggested in HD patients, we assessed the relationship between BMI and chorea. BMI was related to the chorea score in the univariate analysis (β = −0.15, *p* = 0.03). In the multivariate analysis, however, only GH was significantly associated with BMI, independently from the chorea score (β = −0.21, *p* = 0.003).

## Discussion

**Figure 1:**
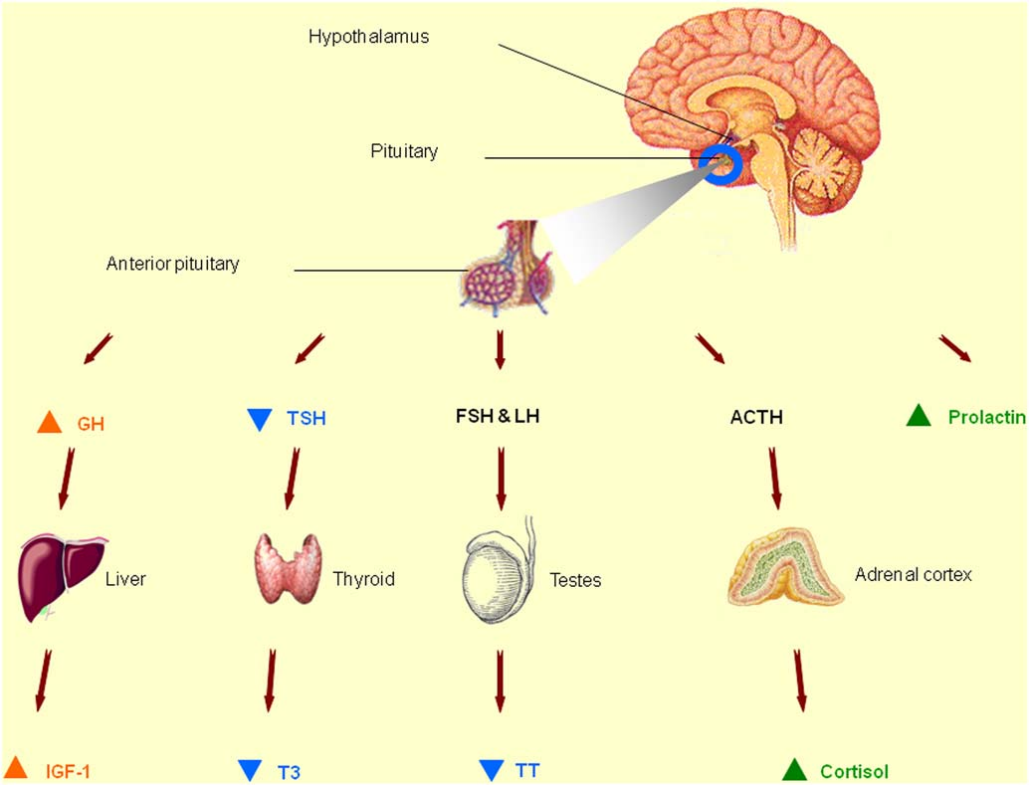
Levels of pituitary and peripheral hormones according to HD stage and comparatively to controls. An orange arrow: early increase in hormone concentration compared to controls. A green arrow: increase in hormone concentration related to an exogenous factor (neuroleptic treatment or stress). A blue arrow: late decrease in hormone concentration related to disease severity. GH, growth hormone; IGF-1, insulin-like growth factor-I; TSH, thyroid stimulating hormone; FT3, free triiodothyronine; ACTH, adrenocorticotropic hormone; LH, luteinizing hormone; FSH, follicle-stimulating hormone; TT, testosterone.

Endocrine disturbances in HD and their link with disease severity have not been investigated previously in a large matched case-control study, despite their pathophysiological and clinical relevance [28]. We demonstrated impairments of several anterior pituitary axes in HD patients (Figure 1). Both central (GH) and peripheral (IGF-1) somatotropic hormones were higher in the patients than in the healthy controls and increased with disease severity. Among corticotropic-axis hormones, only cortisol was increased. In contrast, the thyrotropic-axis and, in men, gonadotropic-axis hormones were decreased with disease severity. The prolactin was increased in patients with neuroleptic treatment. Of the five axes, only the somatotropic axis was related to weight loss.

Both typical and atypical neuroleptics treatment influence hormonal levels of prolactin and GH by altering their dopaminergic regulation [29]. To avoid the biais of neuroleptic treatment, firstly we adjusted on neuroleptics treatment in our multivariate regression models. After, we compared hormonal levels in controls and patients with and without neuroleptics treatment. Using this approach, we found that the prolactin increase in the HD group compared to the matched control group was entirely ascribable to neuroleptic use. On the contrary, neuroleptics blunted partially the high significant difference in GH levels between patients and controls in the overall population. Indeed, this significant difference in GH was higher in non-users group then in group taking neuroleptics.

Basal plasma cortisol was higher in HD patients than in controls, in keeping with earlier studies [17], [18]. This increase was independent from ACTH levels and from disease severity. The dissociation of cortisol levels from ACTH levels suggests a role for other factors in the cortisol increase seen in the patients. An earlier study showed a blunted ACTH response to exogenous corticotropin-releasing hormone [18]. Presumably, chronic stress may be associated with alteration in the hypothalamo-pituitary axes and particularly contributes to increase the cortisol levels [30] in the patients, as seen in other chronic diseases such as schizophrenia [31] and depression [32].

Alterations in sexual behavior have been reported in patients with HD [33]. Our hormone level data fail to provide convincing explanations to these alterations, as plasma testosterone levels were not significantly different between HD patients and controls. An earlier study showed lower testosterone levels in males with HD compared to healthy controls [20]. However, the patients with stage I or II disease had normal testosterone levels, and testosterone levels showed a negative correlation with disease severity [20]. Patients with stage I and II disease contributed 69% of our study population and patients with stage IV or V only 9%. In keeping with the earlier study [20], we found that testosterone levels declined with disease severity. A transgenic mouse model of HD (the R6/2 model) is characterized by atrophy of the testes and infertility [34], which may be related to loss of hypothalamic neurons producing gonadotropin-releasing hormone (GnRH) [35]. The reduction in plasma testosterone levels in our patients was not associated with decreases in FSH and LH, indicating a role for loss of the direct neuronal hypothalamic-testicular pathway in patients with advanced disease.

TSH levels in our patients did not differ significantly from those in the controls but declined with disease progression, in keeping with a previous study [19]. Since, none of the thyroid hormones in our study differed between patients and controls, the declining TSH levels suggest loss of hypothalamic neurons. However, additional studies are needed to clarify this point.

Plasma GH levels were higher in patients than controls, in accordance with previous reports [9], [12]. A few studies conducted in smaller numbers of patients found no increase in GH [10], [16], [36] or IGF-1[37]. However, we found increases not only in GH, but also in the GH effector (free IGF-1). Furthermore, this increased somatotropic activity was associated with disease severity. Importantly, weight loss was significantly related to GH elevation and was independent from motor disorders and other endocrine disturbances. GH deficiency is associated with obesity and GH treatment decreases the fat mass by inducing lipolysis within fat tissue [38]. Thus, increased GH release may be related to the weight loss often seen in HD patients [24].

Concerning IGF-1, the present finding extend and reinforce the observations that level of circulating IGF-1 is altered in many type of human neurodegenerative disease. In HD, it may exert a neuroprotective role by activating the enzyme serine/threonine kinase Akt [21], which phosphorylates the mutant huntingtin protein at serine 421. A protective effect of IGF-1 has been suggested in other neurodegenerative diseases [39]. As well, this elevation of IGF-1 level reflect a resistant state [40] and it is likely due to a loss of sensitivity of target cell to the action of growth hormone.

Therefore, prospective study is necessary to determine whether GH increase is the cause or the result of weight loss and other impairment and to verify if the IGF-1 elevation seen in our patients could reflect an adaptive response to cell death.

In conclusion, our data advocate several neuroendocrine abnormalities in HD. These alterations, although possibly non-specific, may shed light on some of the pathophysiological mechanisms involved in disease progression. Although neuroendocrine dysfunction may contribute to peripheral symptoms such as weight loss, its link to disease progression remains to be confirmed.

## Materials and Methods

### Participants

We enrolled 219 patients with HD characterized genetically by a CAG repeat number greater than 38. These patients were recruited at six centers (Créteil, Paris, Marseille, Strasbourg, Lille, and Lyon) belonging to an HD network (Réseau Huntington de Langue Française, RHLF). The 108 females and 111 males with HD had an average age of 52±10·8 years (range 25–85). They were compared to 71 healthy controls (35 females and 36 males) recruited among spouses or close relatives of the patients and matched to the patients on age and sex, two factors that affect hormone production. The control group of spouses and close relatives is needed to have the most identical group in all relevant ways or in all items (genetics, social, environmental…) to our patients except for the disease in order to eliminate any factors that alter the hormonal states. The control group had an average age of 56·3±13 years (range 26–84).

We matched our patients and controls in a ratio of 3/1 respectively on both age and sex. For age, the interval of matching was 1 to 3 years except for 3 patients and 2 controls who had an interval of 7 to 10. This difference in matching strategy is not anticipated to affect our results, given that it involves a limited number of samples and the differences in hormone levels over a 10 year period is still anticipated to be negligible.

The controls were not at risk for HD and were free from neurological disease. The study protocol was approved by the Henri Mondor Ethics Committee (Créteil, France). Before inclusion, written informed consent was obtained from all patients and controls.

HD patients were assessed by the neurologist of the relevant center. The assessment included a clinical examination using the Unified Huntington's Disease Rating Scale (UHDRS) [1], a medical history, and a questionnaire on past and current symptoms.

The UHDRS is divided into four components that assess motor performance, cognition, behavior, and functional capacity [41]. The motor score evaluates various features including chorea, dystonia, and oculomotor function. The behavioral score measures the frequency and severity of psychiatric symptoms.The functional assessment comprises three sub-scales: the functional checklist (range from 25 to 50), the Independence Scale (IS, range 0 to 100), and the Total Functional Capacity scale (TFC, range 0 to 13). The TFC distinguishes five stages, from I (slightly impaired) to V (severely impaired) [42]. The cognitive assessment was evaluated in only 98 patients and it comprises three tests: Stroop Interference test, Symbol Digit Modalities Test and the Verbal Fluency Test.

Body mass index (BMI) was calculated as weight in kilograms divided by height in meters squared. Medications were recorded, including neuroleptics, antidepressants, tranquillizers, and other drugs without known effects on plasma hormone levels. Three patients were treated for hypothyroidism and were excluded from the analysis of thyroid axis function. We also excluded 2 more patients from all analysis since they had an age of onset inferior to 20 years old.

### Hormone assays

Hormones produced by the five pituitary axes were assayed at a central laboratory: somatotropic-axis hormones (growth hormone [GH], insulin-like growth factor-1 [IGF-1], and insulin-like factor binding protein-3 [IGFBP3]), thyrotropic-axis hormones (thyroid stimulating hormone [TSH], free triiodothyronine [FT3], and free total thyroxine [FT4]), corticotropic-axis hormones (adrenocorticotropic hormone [ACTH] and cortisol), gonadotropic-axis hormones (luteinizing hormone [LH], follicle-stimulating hormone [FSH], and testosterone in men), and prolactin.

Blood samples were drawn in the morning after an overnight fast for all individuals and stored at −80°C until used. Assays were performed according to the manufacturer's instructions for the relevant kit. All samples were tested in a single run using a single reagent lot. GH was assayed using ACCESS2 (Beckman-Coulter, Villepinte, France); TSH, FT3, FT4, FSH, LH, prolactin, testosterone, cortisol, and ACTH using ELECSYS2010 (Roche Diagnostics, Meylan, France); and IGF-1 and IGFBP3 using IMMULITE2500 (DPC, La Garenne Colombes, France).

### Statistical analysis

Arithmetic means with their standard deviation (SD) were computed for normally distributed variables. Variables whose distribution was not normal (GH, TSH, ACTH, prolactin, LH, FSH, and testosterone) were normalized by logarithmic or square-root transformation, and their geometric means with the 95% confidence intervals (95%CI) were computed. Since the normal range of IGF-1 change according to age, all statistical analyses were performed on SDS IGF-1 for IGF-1 concentration. We calculated standard deviation score (SDS) of IGF-1 for each individual according to the following formula [43]:
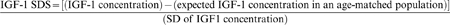



In addition, because IGFBP3 is the main binding protein for IGF-1 and regulates its activity, the IGF-1 level was adjusted for IGFBP3.

With our sample size and the 3∶1 ratio of patients over controls, power was greater than 90% for detecting a statistically significant difference of about 20% between mean hormone levels in patients and controls.

We used t-tests or chi-squared tests to compare patients and controls. The multivariate analysis adjusted for age, sex, and variables yielding significant results in the univariate analyses (BMI, neuroleptic use, and IGFBP3). Since neuroleptics may influence the levels of some hormones, we first adjusted for neuroleptic use in overall analyses and we then compared HD users of neuroleptics to HD non-users and to their controls. Since hormone levels were not influenced by the use of antidepressants or tranquillizers, these variables were not entered into the model (data not shown).

Linear regression analysis was performed to evaluate associations between hormone levels and clinical characteristics. Adjustments were done for age, sex, BMI, neuroleptic use, and IGFBP3, which were significantly related to hormone levels in the univariate analyses.

Differences were considered statistically significant when *p*<0.05. All analyses were conducted using the SPSS 13.0 for Windows package (SPSS Inc, Chicago, IL).
